# Robust expression of tumor suppressor miRNA’s let-7 and miR-195 detected in plasma of Saudi female breast cancer patients

**DOI:** 10.1186/s12885-017-3776-5

**Published:** 2017-11-28

**Authors:** Amal Qattan, Haya Intabli, Wafa Alkhayal, Chafica Eltabache, Taher Tweigieri, Suad Bin Amer

**Affiliations:** 1 Breast Cancer Research, Department of Molecular Oncology, King Faisal Specialist Hospital and Research Centre, P.O.Box 3354, Riyadh, 11211 Saudi Arabia; 2 Department of Biochemistry and Molecular Medicine, School of Medicine and Health Sciences (SMHS), George Washington University, 2600 Virginia Avenue, NW, Suite 300, Washington, DC 20037 USA; 3 College of Medicine, Alfaisal University, P.O.Box 50927, Riyadh, 11533 Saudi Arabia; 4 College of Medicine, Princess Nourah Bint Abdulrahman University, Riyadh, Saudi Arabia; 5 Department of Surgery, King Faisal Specialist Hospital and Research centre, Riyadh, Saudi Arabia; 6 Department of Oncology, King Faisal Specialist Hospital and Research centre, Riyadh, Saudi Arabia

**Keywords:** Circulating miRNAs, Triple-negative breast cancer (TNBC), Circulating biomarkers, Plasma versus tissue, Secretion, FASN pathway, ROC curves, Cancer therapy

## Abstract

**Background:**

Female breast cancer is frequently diagnosed at a later stage and the leading cause of cancer deaths world-wide. Levels of cell-free circulating microRNAs (miRNAs) can potentially be used as biomarkers to measure disease progression in breast cancer patients in a non-invasive way and are therefore of high clinical value.

**Methods:**

Using quantitative RT-PCR, circulating miRNAs were measured in blood samples collected from disease-free individuals (*n* = 34), triple-negative breast tumours (TNBC) (*n* = 36) and luminal tumours (*n* = 57). In addition to intergroup comparisons, plasma miRNA expression levels of all groups were analyzed against RNASeq data from cancerous breast tissue via The Cancer Genome Atlas (TCGA).

**Results:**

A differential set of 18 miRNAs were identified in the plasma of breast cancer patients and 10 miRNAs were uniquely identified based on ROC analysis. The most striking findings revealed elevated tumor suppressor let-7 miRNA in luminal breast cancer patients, irrespective of subtype, and elevated miR-195 in plasma of TNBC breast cancer patients. In contrast, hsa-miR-195 and let-7 miRNAs were absent from cancerous TCGA tissue and strongly expressed in surrounding non-tumor tissue indicating that cancerous cells may selectively export tumor suppressor hsa-miR-195 and let-7 miRNAs in order to maintain oncogenesis.

**Conclusions:**

While studies have indicated that the restoration of let-7 and miR-195 may be a potential therapy for cancer, these results suggested that tumor cells may selectively export hsa-miR-195 and let-7 miRNAs thereby neutralizing their potential therapeutic effect. However, in order to facilitate earlier detection of breast cancer, blood based screening of hsa-miR-195 and let-7 may be beneficial in a female patient cohort.

**Electronic supplementary material:**

The online version of this article (10.1186/s12885-017-3776-5) contains supplementary material, which is available to authorized users.

## Background

The general consensus for breast cancer prevention and treatment includes periodic breast cancer screening of all women and the frequent monitoring of women at higher risk [1]. Nevertheless, cancer statistics indicate that as of January 2017 female breast cancer is the most frequently diagnosed cancer [2]. Mammography is the current gold standard for breast cancer screening and is associated with significant discomfort which impedes early detection [3]. Therefore, finding non-invasive, safe, relatively inexpensive and accurate breast cancer tumor markers [4] as well as potential blood-based biomarkers for the diagnosis and prognostics of breast cancer [5–9] remain important research objectives.

MicroRNAs (miRNAs) are short non-coding RNAs which function as post-transcriptional regulators of gene expression through targeted binding [10–12]. While tissue biomarkers have been extensively studied in cancer detection, circulating miRNAs in body fluids, especially blood serum and plasma, are a promising source of stable non-invasive biomarkers [13, 14]. The tumor specific de-regulation of some miRNAs and their target genes is frequently observed [15, 16] rendering some miRNAs as potential biomarkers for the diagnosis of cancer [13, 17, 18].

Despite the increase in the number of breast cancer biomarker studies, due to the inconsistency of the results, no consensus has been reached on the diagnostic use of differentially regulated circulating miRNAs reported so far [19]. Inconsistencies among studies may be due to patient heterogeneity; genetic background, gender age, metabolic status, as well as methodological challenges [19–24] such as sample size, the number of miRNAs studied, blood collection practices and isolation methods [22]. Moreover, comorbidities such as obesity and diabetes can significantly affect plasma miRNA levels of putative cancer biomarkers [25, 26]. Lastly, the miRNA detection method used, sample type tested (plasma versus serum), and the use of either spike-in or endogenous controls for normalization are the major determinants of study outcomes, regardless of the pathological condition in assessment [24].

Despite these challenges, this work on a purely Saudi, female patient cohort to investigate whether a stable circulating plasma miRNA signature could distinguish between disease-free individuals (*n* = 34) and early stages diagnoses of triple negative breast cancer (TNBC) (*n* = 36) and luminal breast cancer tumor (*n* = 57). Due to the relatively early diagnosis of cancer in this cohort, patient tissue biopsies were not available for analyses. Therefore, in order to compare differences in miRNA expression levels in tumor tissue versus plasma, plasma miRNA expression was compared to publically available RNASeq data from cancerous breast tissue and surrounding non-cancerous tissue available via The Cancer Genome Atlas (TCGA).

## Methods

### Ethical statement

Approval and written consent was obtained from all study participants for the use of their blood samples for research purposes. The study was approved by the Ethical Research Committee and Basic Research Committee on Clinical Research at KFSHRC, Riyadh, Saudi Arabia and was carried out under the terms of the Helsinki Declaration.

### Study cohort and clinical samples processing

A total of 127 females, disease-free individuals (*n* = 34), triple-negative breast tumors (TNBC) (*n* = 36) and patients with luminal tumors (*n* = 57) were recruited for the study. All women were of Saudi background and recruited at the King Faisal Specialist Hospital (KFSHRC). Details of the study subjects with respect to the age of diagnosis, grade and lymph node status are reported in (Additional file 1: Table S1). All blood samples were obtained from patients before any cancer therapy was administered. Blood samples were collected by vene-section in EDTA blood collection tubes (BD Vactainer, Plymouth, UK) and kept at 4 °C. The blood was then centrifuged within 2 h at 1500 g at 4 °C for 15 min to isolate the plasma. The plasma was collected and centrifuged again at 2500 g at 4 °C for 15 min (Heraeus multifug 3S-R-UK) to eliminate the debris. All samples were stored at -80 °C.

### Isolation of microRNA from plasma

RNA was isolated from the plasma using the miRNeasy Serum/Plasma Kit (Qiagen, Hilden, Germany), following the manufacturer’s protocol with some modifications. RNA isolation was performed in duplicate. For the Qiagen kit assay, 1 ml of QIAzol lysis reagent (Qiagen) was added to 200 μl of plasma together with 1 μg of carrier MS2 RNA (Roche). The samples were mixed and incubated for 5 min at room temperature. To monitor the RNA isolation before purification 3.5 μl (1.6 × 10^8^ copies/μl) of *C.elegans*-miR-39 miRNA mimic spike-in control was added and 12 μg of pure glycogen was added as a carrier/co-precipitant. Next, 200 μl of chloroform was added to the starting sample and mixed for 15 s. Next, samples were incubated for 3 min at room temperature, and centrifuged for 15 min at 12,000 g at 4 °C. 600 μl of the aqueous phase was transferred to a new tube and 900 μl of 100% ethanol was added to the spin column to allow all the RNA molecules to reach the binding condition. After mixing, the samples were transferred to RNeasy MinElute spin columns in a 2 ml collection tube and spun for 15 s at ≥8000 g. The columns were washed with two buffers from 700 μl RWT buffer and then with 500 μl RPE buffer (Qiagen, Hilden, Germany), with a short spin of 15 s at ≥8000 g each time. Next, each column was washed with 500 μl of 80% ethanol and centrifuged for 2 min at ≥8000 g. Following 5 min high speed centrifugation, 14 μl of RNase-free water could be used for elution. All RNA samples were frozen at -80 °C until further analysis.

### Assessment of RNA quality and integrity

Quality of RNA was assessed by Nanodrop ND-2000 (Wilmington, DE, USA). Chromatographic characteristics and integrity of all RNA samples were determined, included interpretation of the peak detection of different profiles, by means of RNA 6000 Nano LabChip, (Agilent Technologies, Waldbronn, Germany), Agilent 2100 Bioanalyzer system (Agilent Technologies, Santa Clara, USA) and the 2100 expert software tool (Agilent Technologies, Santa Clara, USA).

### Quantitative real time polymerase chain reaction for mature miRNA expression profiling [13, 27–29]

250 ng of the eluted RNA sample was used to make cDNA using a miScript RT II kit with miScript HiSpec buffer from Qiagen (Qiagen, Hilden, Germany). Briefly, the reaction was set up in the thermal cycler for 60 min at 37 °C followed by 5 min at 95 °C. The cDNA was then diluted with 200 μl of RNase-free water. For RT-qPCR a total of 2750 μl was prepared, made up of (2 x QuantiTect SYBR green PCR master mix, 10 x miScript universal primers with a cDNA template and RNase free water). MicroRNAs screening was performed using miScript miRNA PCR Array Human Breast Cancer-MIHS-109Z (Qiagen, Hilden, Germany). The miRNA PCR Array Panel contains 84 mature miRNAs most relevant to breast cancer tumorigenesis. The final reaction volume was 25 μl per well, enough to provide 1 ng cDNA per well. The plates were run following a thermal cycling protocol: 95 °C for 15 min to activate the HotStar Taq DNA polymerase, 40 amplification cycles of 15 s at 94 °C, 30 s at 55 °C, 30 s at 70 °C and at the end a melting curve program. All qPCR reactions were run in duplicate.

### Data processing and statistical analysis

All samples passed ‘Positive PCR Controls’ (PPC) in which the acceptable range of Ct values was set to 19 ± 2. Both Reverse Transcription Controls (RTC) and (PPCs) were used to assess whether there had been any inhibition during the reverse transcription reaction. Avg Ct^miRTC^ – Avg Ct^PPC^ was calculated for each sample (Avg “average”). A difference of greater than seven indicated impurities and reaction inhibition, cellular contamination was assessed using the mean Ct of the SNORNA (SNORD) controls. Only the non-zero values were considered. SNORD72 was excluded from computations as it performed poorly across samples. A sample with a mean Ct < 32 was taken to indicate cellular contamination. All samples used in the qRT-PCR analysis were tested for quality and neither indication of cellular contamination nor reaction inhibition was detected. All samples collected were retained and none discarded. For miRNA to be within the detection limit, the Ct values were recommended to be between zero and 35. Cel-correction to correct for technical variations that arise during extraction procedure, exogenous spike-in controls from *C.elegans* was used. The Qiagen miScript miRNA PCR Array Human Breast Cancer array panel contains two Cel-miR-39 spiked-in controls. The average Ct value recorded in each sample for the spiked-in controls from *C.elegans*, cel-39 was recorded and the median of average Ct values (of all samples) was found. The normalizing factor for each sample was calculated by subtracting the median of all average Ct^cel^ values from the average Ct^cel^ value for the sample. The ddCt computation (ΔΔCt) for each of the three groups, the triple negative (TNBC) and the luminal tumors, average dCt (ΔCt) was calculated for each miRNA. The miRNAs with average dCt values (ΔCt) below 15 and/or above 35 were excluded. ddCt (ΔΔCt) of a given miRNA for a given pair of groups was computed by finding the difference between the average dCt (ΔCt) of the respective groups; for example, ΔΔCt(ddCt _(Triple Negative vs. Controls))_ = [Average dCt (TNBC)] – [Average dCt (Controls)]**.** The relative expression of a given miRNA between any two groups was assessed by computing 2^(−ddCt)^ (2^(−ΔΔCt)^). A differential set was identified using the ddCt (ΔΔCt) method proposed by Livak et al. [30]. The Mann-Whitney unpaired test and Benjamin-Hochberg multiple testing corrections were used to determine significant differences in miRNA expression levels between groups [31]. All qPCR reactions were run in duplicate.

### Bioinformatics analysis

The miRNA targets and the biological pathways they were involved in were predicted using the microT-CDS algorithm and mirPath v.2.0 available on the web-based server DIANA. The micro-T threshold for target prediction was set at 0.8 and targeted pathways were considered significant at a *p-value* < 0.05 [32, 33]. Hierarchical clustering was performed using GeneSpring GX 14.5.

### Receiver operating characteristic (ROC) curves

Receiver Operating Characteristic (ROC) curves were generated using the web-based tool ROCCET [34] for finding two sets of miRNAs that could best differentiate (i) triple-negative tumour samples from normal (control) samples and (ii) luminal tumour samples from normal (control) samples. ROC (Receiver Operating Characteristic) curves were then generated by Monte-Carlo Cross Validation (MCCV). The procedure was performed repeatedly to calculate the performance and confidence interval of each model.

### Comparison of miRNA levels in tissue and plasma

Since plasma levels of miRNA are not necessarily a reflection of tissue levels [35–38] and tumor tissue was not available from patients recruited for this study as plasma was collected pre-cancer therapy, publicly available data from the TCGA (The Cancer Genome Atlas) [39] was used to determine whether circulating plasma levels were distinct from breast cancer tissue and surrounding non-cancerous tissue levels. We compared the observed miRNA plasma levels with the tissue levels of corresponding miRNA precursors from the TCGA study. The tissue level expression of miRNA precursors, available as RPKM (reads per kilobase of transcript per million) values was obtained for control samples (*n* = 87), luminal samples (*n* = 120) and Triple Negative samples (*n* = 38) from TCGA. The RPKM values of the precursors in tissue and the expression value for miRNAs in the current study were log_2_ transformed and auto-scaled, to ensure the datasets are comparable.

$$ \mathrm{Auto}\  \mathrm{scaled}\  \mathrm{value}=\left(\mathrm{x}-\upmu \right)/\updelta $$

The tissue RPKM and the plasma cel-corrected C_t_ values are normalized to the mean (μ) and standard deviation (δ) for each of the data. Since a miRNA precursor can give rise to an active form from each arm, we compared both the 3′ and 5′ active forms were matched to the same precursor. The 18 differentially expressed active miRNA forms mapped to 17 precursor miRNAs from TCGA. Then we analyzed the expression trends of a given miRNA across tissue and plasma samples.

## Results

### Differences in circulating miRNAs between breast cancer patients and normal samples

**Fig. 1:**
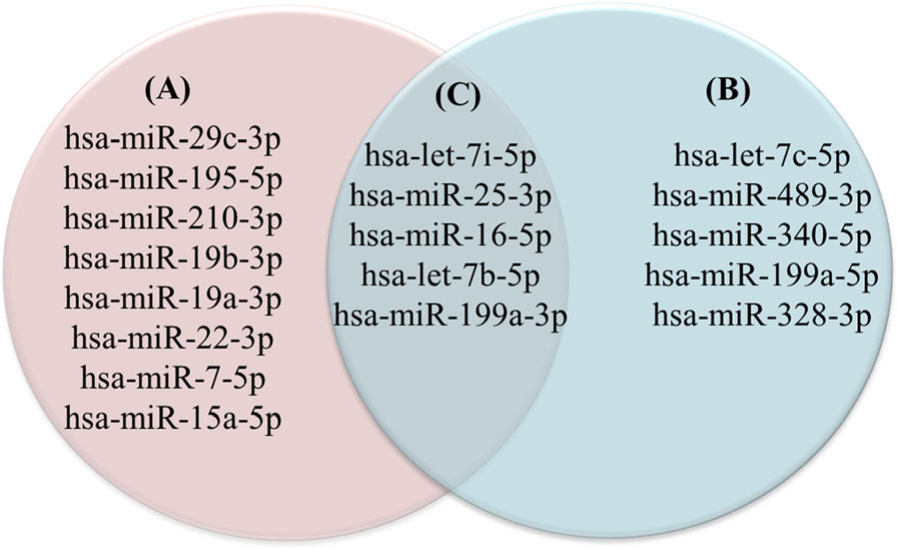
Circulating miRNAs showing differential levels in breast cancer patients (Triple negative and Luminal) with respect to disease-free individuals. Three main groups: TNBC vs. healthy controls (Group A; eight significantly regulated miRNAs); luminal patients vs. healthy controls (Group B; five significantly regulated miRNAs); and breast cancer patients irrespective of subtype and healthy controls (Group C; five significantly regulated miRNAs)

Comparative analysis identified an initial set of 18 circulating miRNAs (Table 1), which because of their differential presence between the patient groups and healthy controls, were further examined by cancer type. Figure 1 illustrates relative expression of these 18 miRNAs subdivided into three groups: TNBC plasma vs. disease free plasma (Group A; *n* = 8 miRNAs: hsa-miR-29c-3p, hsa-miR-195-5p, hsa-miR-210-3p, hsa-miR-19b-3p, hsa-miR-19a-3p, hsa-miR-22-3p, hsa-miR-7-5p, hsa-miR-15a-5p); luminal patient plasma vs. disease free plasma (Group B; *n* = 5 miRNAs: hsa-let-7c-5p, hsa-miR-489-3p, hsa-miR-340-5p, hsa-miR-199a-5p, hsa-miR-328-3p); lastly, all breast cancer patients (irrespective of subtype) vs. disease free plasma (Group C; n = 5 miRNAs: hsa-let-7i-5p, hsa-miR-25-3p, hsa-miR-16-5p, hsa-let-7b-5p, hsa-miR-199a-3p).

**Table 1:** Fold change (FC) and *p-values* of the 18 significant miRNA

	Triple Negative				Luminal			
miRNA ID	2^-(TNBC-C)^A^	p (Corr) TNBC vs C^P^	Regulation (TNBC vs C)	FC (TNBC vs C)^F^	2^-(L-C)^B^	p (Corr) L vs C ^P^	Regulation (L vs C)	FC (L vs C)^F^
hsa-let-7b-5p	2.14	**0.006**	Up	**2.14**	2.29	**0.000**	Up	**2.29**
hsa-let-7c-5p	1.72	0.031	Up	**1.72**	1.95	**0.001**	Up	**1.95**
hsa-let-7i-5p	1.71	**0.008**	Up	**1.71**	1.60	**0.008**	Up	**1.60**
hsa-miR-15a-5p	2.01	**0.006**	Up	**2.01**	0.91	0.977	Up	−1.09
hsa-miR-16-5p	2.20	**0.000**	Up	**2.20**	1.68	**0.008**	Up	**1.68**
hsa-miR-195-5p	1.84	**0.004**	Up	**1.84**	1.60	0.011	Up	**1.60**
hsa-miR-199a-3p	0.36	**0.002**	Down	**−2.81**	0.36	**0.003**	Down	**−2.80**
hsa-miR-199a-5p	0.46	0.021	Down	**−2.19**	0.25	**0.001**	Down	**−3.94**
hsa-miR-19a-3p	1.81	**0.006**	Up	**1.81**	1.29	0.205	Up	1.29
hsa-miR-19b-3p	1.86	**0.006**	Up	**1.86**	1.27	0.203	Up	1.27
hsa-miR-210-3p	1.91	**0.000**	Up	**1.91**	1.23	0.646	Up	1.23
hsa-miR-22-3p	1.93	**0.006**	Up	**1.93**	1.08	0.083	Up	1.08
hsa-miR-25-3p	2.02	**0.001**	Up	**2.02**	2.01	**0.001**	Up	**2.01**
hsa-miR-29c-3p	1.66	**1.010**	Up	**1.66**	1.19	0.203	Up	1.19
hsa-miR-328-3p	1.58	0.563	Up	**1.58**	20.96	**0.000**	Up	**20.96**
hsa-miR-340-5p	0.65	0.043	Down	**−1.54**	0.55	**0.008**	Down	**−1.81**
hsa-miR-489-3p	1.85	0.057	Up	**1.85**	2.26	**0.000**	Up	**2.26**
hsa-miR-7-5p	2.30	**0.003**	Up	**2.30**	2.01	0.012	Up	**2.01**

*TNBC* Triple Negative Breast Cancer

C - Disease-free individuals used as Controls

L - Luminal Breast Cancer

^A^: 2^-(TNBC-N) represents 2^ddCt values of Triple Negative patient’s samples as compared to those of disease-free individuals

^P^: Corrected p-values ≤ 0.01 for a given pair of conditions are shown in bold

^F^: Fold change values ≥ 1.5 are shown in bold

^B^: 2^-(L-N) represents 2^ddCt values of Luminal patients’ samples as compared to those disease-free individuals

### Comparison of circulating miRNAs levels with cancerous and non-cancerous tissue expression

**Fig. 2:**
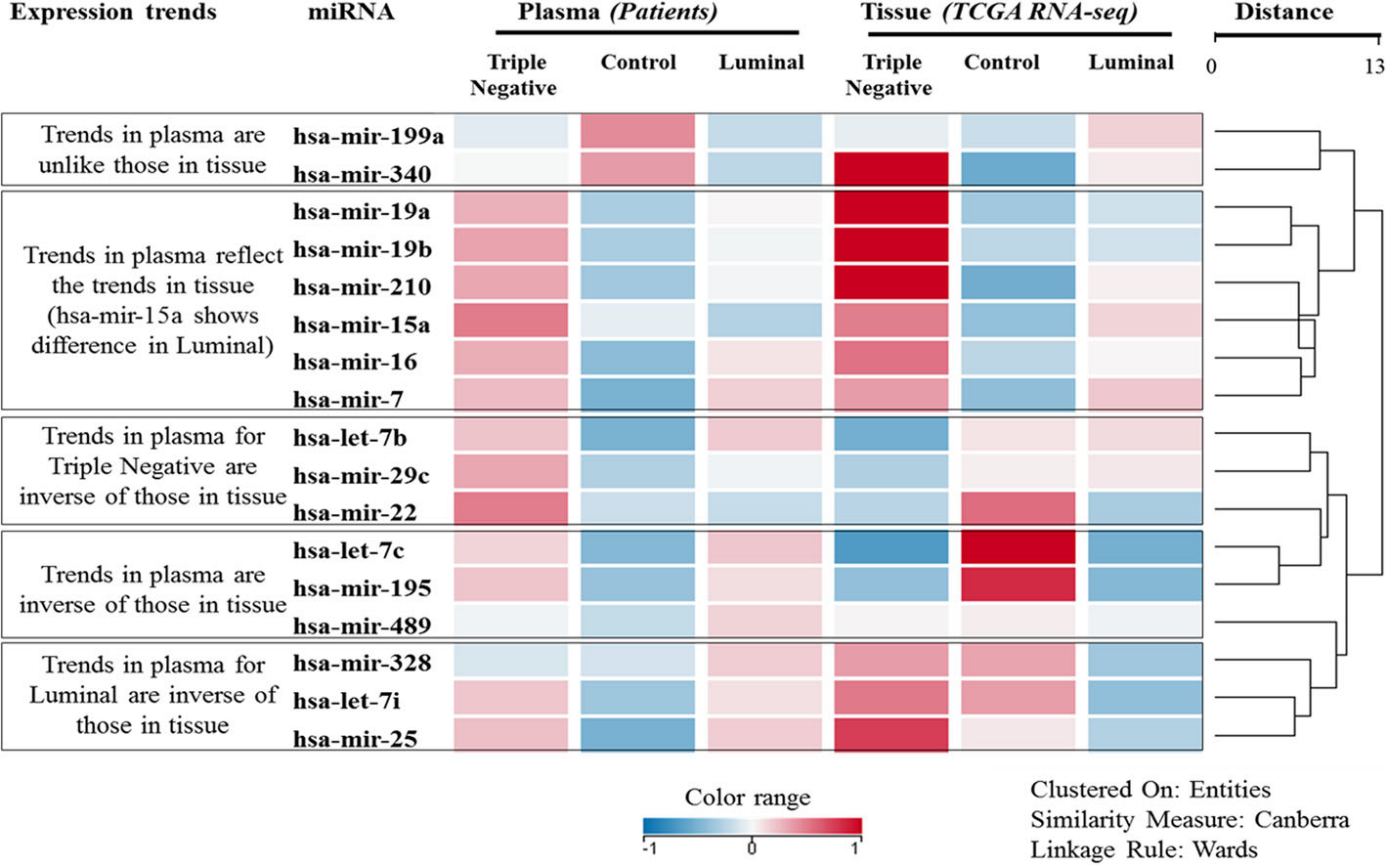
Comparison of miRNA levels in tissue and plasma. Hierarchical clustering view of the normalized expression levels of active forms of miRNA. The tissue level expression of miRNA precursors available as RPKM (reads per kilobase of transcript per million) values were obtained from TCGA RNASeq data as follows: control samples (*n* = 87), Triple negative breast cancer samples (*n* = 38) and Luminal samples (*n* = 120). The RPKM values of the precursor in tissue and the expression value in the current qPCR (inferred as 2-dCt (2(−ΔCt))) for circulating miRNA, were log2 transformed and auto-scaled to ensure the data are comparable

Next, the tissue expression trends of the corresponding 17 miRNA precursors in relation to their active forms in plasma were measured (Fig. 2). Since plasma collected from all patients enrolled in this study occurred prior to chemotherapy administration and/or tumor biopsy, miRNA breast cancer tissue and non-cancerous tissue expression values were obtained from publically available TCGA RNASeq data; non-cancerous tissue samples (*n* = 87), luminal samples (*n* = 120) and Triple Negative samples (*n* = 38). Expression trends for some miRNAs (hsa-miR-19a, hsa-miR-19b, hsa-miR-210, hsa-miR-16, hsa-miR-7 and to a certain extent hsa-miR-15a) were similar in both tissue and plasma. Tissue and plasma levels of non-diseased controls compared against both cancer patient groups showed a broad reversal of the trend. For example, tumor repressor miRs hsa-let-7c and hsa-miR-195 were significantly decreased in both luminal and TNBC breast cancer tissue levels (TCGA) and increased in non-tumor tissue samples. Slight variations of this pattern were observed for hsa-miR-489, hsa-miR-328, hsa-miR-25, hsa-let-7i, hsa-let-7b, hsa-miR-29c, hsa-miR-199a, hsa-miR-340 and hsa-miR-22.

### Pathway analysis of target genes

Lastly, a pathway analyses was performed on the target genes of the 18 differential miRNAs identified in this study (Additional file 1: Figure S1). Not surprisingly, many of them were involved in signaling functions, namely the PI3K-Akt, mTOR, p53, TGF-beta, Wnt, FoxO, estrogen signaling and Hippo signaling pathways. However, the most significantly enriched pathways were the ECM-receptor interaction (Extracellular Matrix) and the fatty acid biosynthesis (FASN) pathways. Gene targets of the let-7 family were found to be involved in ECM receptor interaction while the fatty acid biosynthesis pathway (FASN) was shown to be enriched mainly by hsa-miR-16-5p, hsa-miR-15a-5p and hsa-miR-195-5p. Both hsa-miR-15a-5p and hsa-miR-195-5p were enriched exclusively in the plasma of TNBC patients (see Fig. 1).

### Receiver operating characteristic (ROC) curves

**Fig. 3:**
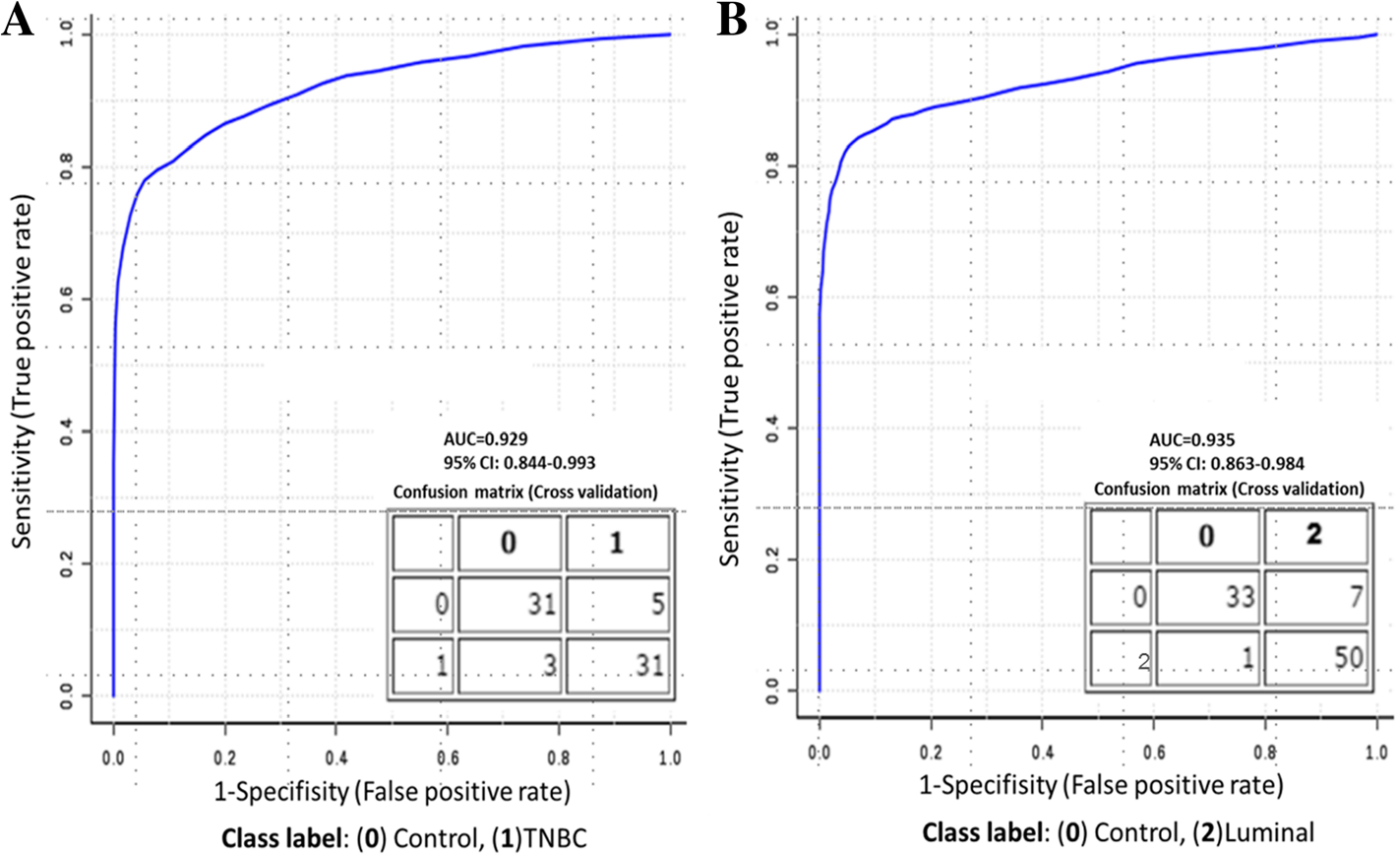
Receiver-operating characteristic (ROC) curve analyses **(a)** Panel of 7 miRNAs consisting of hsa-miR-199a-3p, hsa-miR-15a-5p, hsa-let-7c-5p, hsa-miR-7-5p, hsa-miR-195-5p, hsa-miR-489-3p and hsa-let-7i-5p showed the maximum discriminatory potential between triple negative tumors and disease-free individuals. Similarly, a five miRNA **(b)** Panel consisting of hsa-miR-328-3p, hsa-miR-199a-3p, hsa-let-7i-5p, hsa-miR-195-5p and hsa-miR-25-3p best differentiated luminal tumors patients from the disease-free individuals

In order to assess the potential of each of these miRNAs as cancer biomarkers, we generated ROC curves. Since Univariate AUC ROC curves looked promising, whether a more robust prediction could be made using multiple miRNAs was explored. A panel of seven miRNAs consisting of hsa-miR-199a-3p, hsa-miR-15a-5p, hsa-let-7c-5p, hsa-miR-7-5p, hsa-miR-195-5p, hsa-miR-489-3p and hsa-let-7i-5p showed the maximum discriminatory potential between TNBC patient plasma and disease-free plasma (Fig. 3a). Similarly, a panel of five miRNAs consisting of hsa-miR-328-3p, hsa-miR-199a-3p, hsa-let-7i-5p, hsa-miR-195-5p and hsa-miR-25-3p best predicted luminal tumor patients from disease-free individuals (Fig. 3b). The Univariate AUC statistic for each miRNA is provided (Additional file 1: Table S2).

## Discussion

In less developed countries, including those of the Middle East, breast cancer accounted in 2012 for 25% of all reported cancer cases in females. It has been estimated that in 2020 more than 1.9 million women will be diagnosed with breast cancer, marking an increase of 18.4% [40]. Given the expected increase in female breast cancer diagnosis, the aim of this study was to discover whether any putative circulating miRNA biomarkers, could be differentially detected in the plasma of early stage, treatment-naïve female breast cancer patients. Analyses were performed on plasma isolated from healthy, cancer-free females (*n* = 34), cancer therapy naïve patients diagnosed with triple-negative breast cancer tumors (TNBC) (*n* = 36) and finally, cancer therapy naïve patients with luminal tumors (*n* = 57). As patient tumor biopsies were not available at the time of plasma collection, plasma miRNA expression levels in cancer groups were analyzed not only against the plasma of healthy but also against publically available RNASeq data from non-cancerous tissue samples (*n* = 87), luminal samples (*n* = 120) and triple negative samples (*n* = 38), provided by the (The Cancer Genome Atlas (TCGA)).

It is exceptionally challenging to discuss the results of miRNA biomarker studies in the context of the literature as reports are very contradictory. A meta-analyses performed by Leidner et al. [41] demonstrated major inconsistencies in qPCR as well as genome-wide approaches for detecting miR biomarkers. For example, with the exception of miR-155 and miR-21, none of the 25 miRNAs analyzed by qPCR by eight independent groups; whose cohort sizes were similar to the one used in this study, were detected to be in agreement by more than one study. Furthermore, the findings of significantly elevated circulating miR-155 and miR-21 by qPCR in breast cancer were actually contradicted by subsequent data reported by genome-wide approaches leading to what Leidner refers to as a dampening of enthusiasm for miRNA biomarkers [41]. However, the relatively pure genetic background of the patient population may increase the likelihood of reproducibility as well as the possibility for clinical biomarker application.

As described by Witwer et al. [19] and Chen et al. [42], the composition of circulating miRNAs in cancer patients is governed by the following: 1) active secretion and/or passive leakage of miRNA from tumor cells, 2) increased cellular production and secretion, 3) enhanced selective secretion, and 4) changes in miRNA stability. Similarly, down-regulation of miRNA in the plasma may indicate reduced secretion, increased retention and/or possibly represent a general neoplastic state [19]. For these reasons as well as the fact that biopsies from chemotherapy naïve patient were not available, the differentially regulated miRNAs identified in the plasma samples in this study were compared with tissue levels of miRNA precursors from The Cancer Genome Atlas (TCGA) (Fig. 2).

This analysis performed on this patient cohort led to the identification of three broad categories representing distinct expression patterns of miRNAs. The first category consists of hsa-miR-19a, hsa-miR-19b, hsa-miR-210, hsa-miR-15a, hsa-miR-16 and hsa-miR-7 which are overexpressed in TNBC tissues as well as plasma. Therefore, these miRNAs may directly reflect TNBC tumor biology. To date, many studies have confirmed hsa-miR-19a/b oncogenic role in TNBC tumor biology by repressing PTEN and activating NF-kB [43] and levels of circulating miR-19 correlated with response to neoadjuvant epirubicin + paclitaxel chemotherapy regimen in Stage II and III patients with luminal A breast tumors [44]. In a Japanese TNBC patient cohort, high hsa-miR-210 expression was identified as an independent factor indicating poor prognosis for TNBC [45]. In contrast, members of the miR-15 family have tumour suppressor properties. Hsa-miR-16-5p and hsa-miR-15a-5p are involved in the cell cycle, differentiation, proliferation, hormone regulation and immune response [46]. Various studies reported their down-regulation in most tumours [47]. However, miR-15 family miRNAs are strongly regulated by hormones [48]. Given their multiple functions and complicated regulation, it is unlikely that miR-15 family members would make an sufficient early biomarker for breast cancer. The second category of miRNAs consisted of hsa-miR-199a and hsa-miR-340 which were differentially regulated in tissue and plasma. In contrast to the first group, these miRNAs were decreased in breast cancer patient plasma compared to healthy controls. The third category has several distinct sub-groups. An inverse pattern between plasma and tissue levels specific to TNBC patients was observed for hsa-let-7b, hsa-miR-29c, and hsa-miR-22. In all cases the plasma miRNAs levels were higher than the tissue levels, supporting evidence of cancer cell secretion of miRNAs.

The main finding of this study is that blood based screening of has-miR-195 and let-7 may help to identify and diagnose early stages of breast cancer patients. Elevated circulating levels of let-7 family members (has-let-7b, has-let-7c and has-let-7i) were observed. Studies using breast cancer cell lines [36] and other cancer cell lines [37] have reported the selective release of tumor suppressor miRNAs into extra-cellular fluids. Therefore, extra-cellular miRNAs are not merely the artefacts excreted by dead tumour cells but key players assisting in tumor development and metastasis by promoting cancer-host cross talk [49]. As illustrated by Falcone et al. [50], tumor cells use a multi-pronged approach to create a metastatic niche by selectively secreting out tumor suppressor miRNAs, thereby overcoming immune surveillance by repressing the immune system and promoting angiogenesis. Various members of the let-7 family have been reported to be down-regulated in cancer tissues. Furthermore, it has been shown that the restoration of let-7 levels in cells could be an effective cancer therapy [51]. Thus it is probable that breast cancer tumour cells selectively secrete tumor suppressor miRNAs to maintain oncogenesis as suggested by Ohshima et al. [35]. Let-7 was also increased in the TNBC patient plasma from an Irish patient cohort [52] while in this population, let-7 was only increased in luminal patients. In contrast, an Indian cohort [53] observed decreased miR-195, and increased Let-7 miRNA in circulating plasma of TNBC patients. These highly variable results may be due to a variety of patient variables such as metabolic status, age, cancer stage; controls used, and may also highlight the influence of genetic background on miRNA expression. At the time of this manuscript revision, patient recruitment is currently ongoing for a robust blinded validation experiment.

Based on the pathway analysis performed in DIANA [32, 33], the 18 miRNAs detected in this study and their targets are extensively involved in FASN pathways, ECM-receptor interaction, PI3K-Akt, mTOR, p53, TGF-beta, Wnt, FoxO, estrogen signaling and Hippo signaling pathways, all critical for carcinogenesis. Chen et al. [54] reported hsa-miR-195-5p as a direct regulator of GLUT3 and the increased amounts of GLUT3 transcripts seem to facilitate accelerated metabolism, high glucose requirements, and increased glucose uptake in malignant cells. Using cell lines, Singh et al. [55] demonstrated the anti-cancer activity of hsa-miR-195 and suggested overexpression of hsa-miR-195 as a potential therapy for breast cancer. In this study, circulating hsa-mir-195 levels in TNBC plasma are higher than those in healthy individuals. Likewise, an increased systemic miR-195 levels was observed in blood of breast cancer patients [52, 56–58]. Non-cancerous TCGA tissue had high levels of tumor suppressor hsa-miR-195 while TNBC cancer tissue had low levels suggesting that hsa-miR-195 is secreted out of cancer cells, possibly to facilitate increased GLUT3 expression.

Finally, in the panel of miRNAs selected for distinguishing both TNBC and luminal patients from healthy controls, the receiver operating characteristic (ROC) analysis consistently included hsa-let-7 and hsa-miR-195. Compared to cancer-free plasma samples, let-7 miRNA was most elevated and associated with luminal breast cancer diagnosis, irrespective of subtype, and miR-195 was elevated in TNBC plasma and most associated with TNBC breast cancer patients. In contrast, hsa-miR-195 and let-7 miRNAs were absent from cancerous TCGA tissue and strongly expressed in surrounding non-tumor tissue indicating that breast cancer tumor cells may selectively export hsa-miR-195 and let-7 miRNAs. Taken together, these observations suggest that any study evaluating the use of the over-expressed hsa-let-7 family and/or hsa-miR-195 as anti-cancer therapy should consider that tumor cell machinery may actively target and excrete hsa-miR-195, thereby neutralizing its anti-cancer effect [59, 60]. However, these miRNAs may be of potential use in the development of a blood based screening test to complement and improve early detection of breast cancer [58].

## Conclusion

Plasma sampling from patients remains the least invasive method for identifying biomarkers so any circulating miRNA with disease specific expression would be advantageous to clinicians. Breast cancer specific expression requires that the putative biomarker expression remains tightly linked to biological changes occurring during the onset of tumor growth and through metastasis. Results from this study suggest that both miR-195 and let-7 make satisfactory candidates for biomarkers. However, since levels of let-7 have also been reported to be increased in the serum/plasma of patients with other types of cancer, a biomarker test alone would not be sufficient to determine a diagnosis. However, let-7 may be a better candidate than other miRs such as miR-15 or miR-29 which are regulated not only by the process of tumorgenesis but also by hormones, which may lead to more variability in results. Furthermore, while some studies have indicated that the restoration of let-7 and miR-195 may be a potential therapy for cancer; this study found that circulating hsa-miR-195 levels in TNBC plasma are already significantly higher than those of healthy individuals. These results also suggest that tumor cells may selectively export hsa-miR-195 and let-7 miRNAs thereby neutralizing their potential therapeutic effect [59, 60]. Finally, the model constructed by ROC of a panel of seven miRNAs showed the maximum discriminatory potential between TNBC patient plasma and disease-free plasma while a panel of five miRNAs best predicted luminal tumor patients from disease-free individuals. Future experiments performed on this patient cohort should confirm findings in plasma, patient tissue, and track these markers through the course of treatment (including tissue from mastectomies) and during remission. While large scale studies are necessary to confirm these results before they can be applied into clinical practice, the miRNAs differentially detected in the plasma of breast cancer patients in this study warrant further investigation.

## Additional files

Additional file 1: Table S1. Characteristics of breast cancer patients. **Figure S1.** Heat map of pathways enriched by the target genes for the 18 differential miRNAs. The signalling pathways, namely PI3K-Akt, mTOR, p53, TGF-beta, Wnt, FoxO, estrogen, Hippo signalling and ECM receptor interaction, fatty acid metabolism and fatty acids biosynthesis pathways are enriched. **Table S2.** Univariate AUC statistics for the differential miRNAs based on ROC Analysis. (DOCX 1154 kb)

## Abbreviations

Ct: Threshold cycle
miRNA: microRNA
qRT-PCR: Quantitative Real Time Polymerase Chain Reaction
RNASeq: Ribonucleic acid sequencing
ROC: Receiver Operating Characteristic
RPKM: Reads Per Kilobase of transcript per Million mapped reads
