# Supplementary material for: Robust expression of tumor suppressor miRNA’s let-7 and miR-195 detected in plasma of Saudi female breast cancer patients
Source: BMC Cancer. 2017 Nov 28;17:799. doi: 10.1186/s12885-017-3776-5 (PMC5706292; doi:10.1186/s12885-017-3776-5)
Supplement: Supplementary file 1 — Characteristics of breast cancer patients. Figure S1. Heat map of pathways enriched by the target genes for the 18 differential miRNAs. The signalling pathways, namely PI3K-Akt, mTOR, p53, TGF-beta, Wnt, FoxO, estrogen, Hippo signalling and ECM receptor interaction, fatty acid metabolism and fatty acids biosynthesis pathways are enriched. Table S2. Univariate AUC statistics for the differential miRNAs based on ROC Analysis. (DOCX 1154 kb) [file 12885_2017_3776_MOESM1_ESM.docx]

**Additional files**


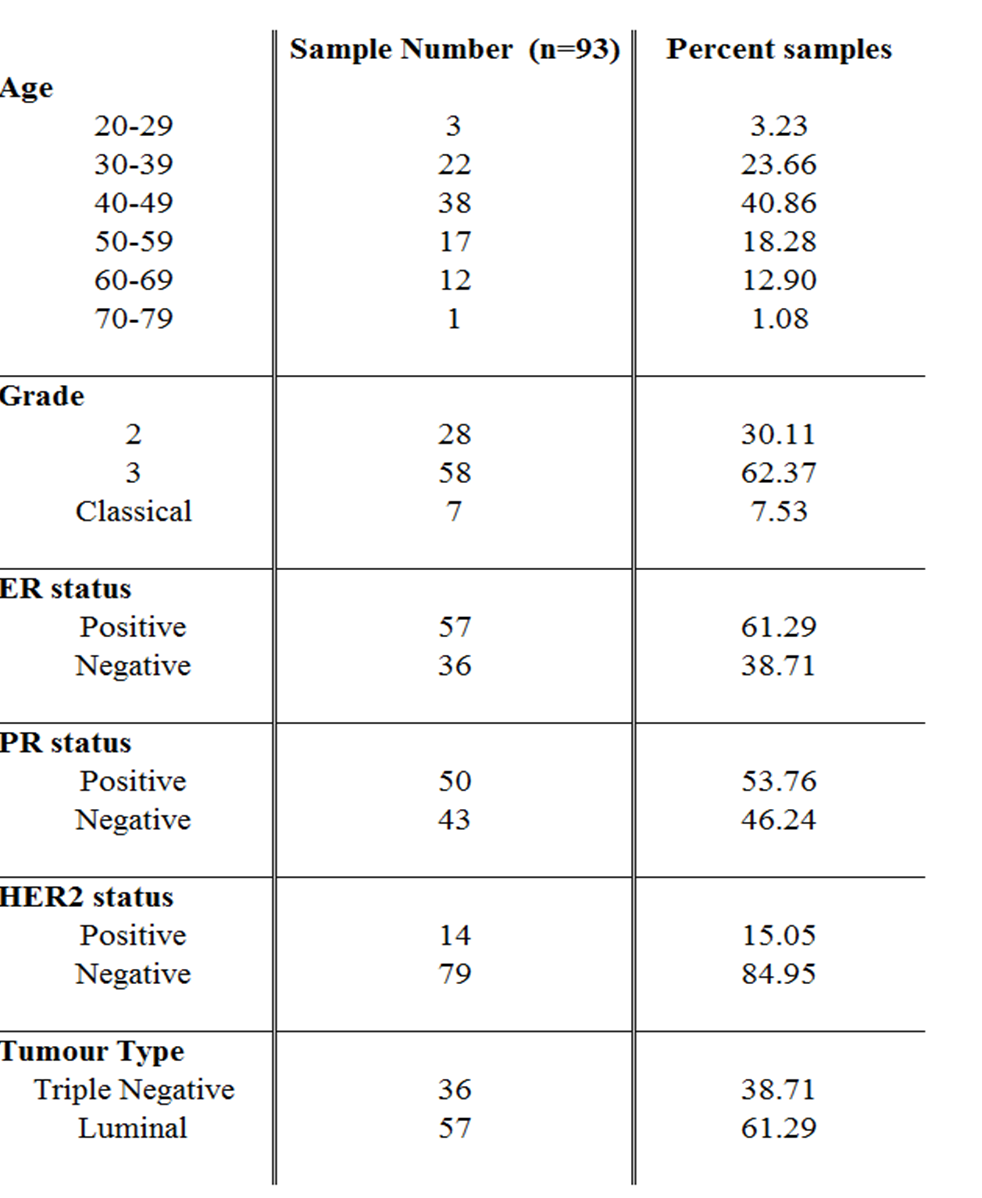


**Additional file 1: Table S1** Characteristics of breast cancer patients


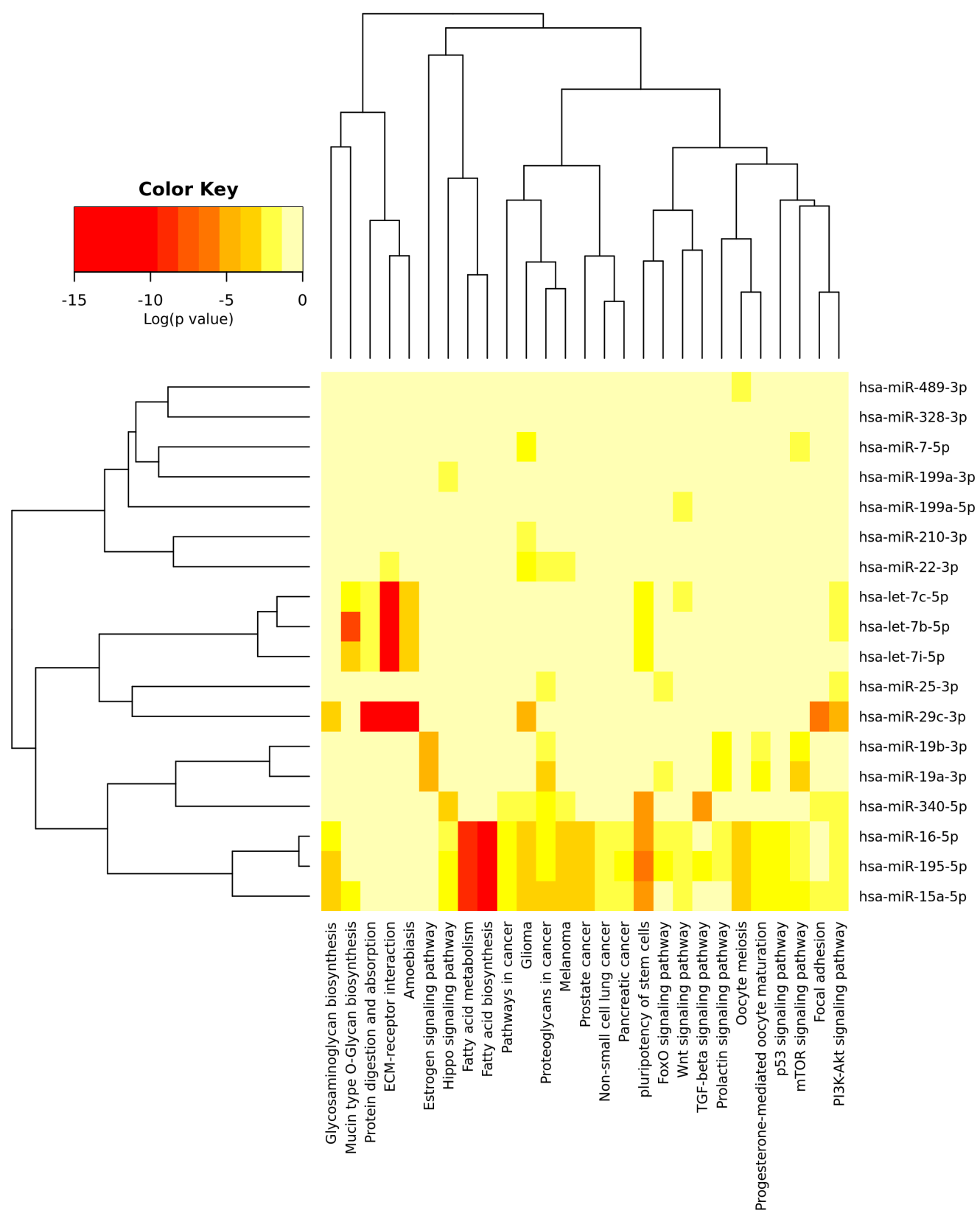


**Additional file 1: Figure S1** Heat map of pathways enriched by the target genes for the 18 differential miRNAs. The signalling pathways, namely PI3K-Akt, mTOR, p53, TGF-beta,Wnt, FoxO, estrogen, Hippo signalling and ECM receptor interaction, fatty acid metabolism and fatty acids biosynthesis pathways are enriched.

**
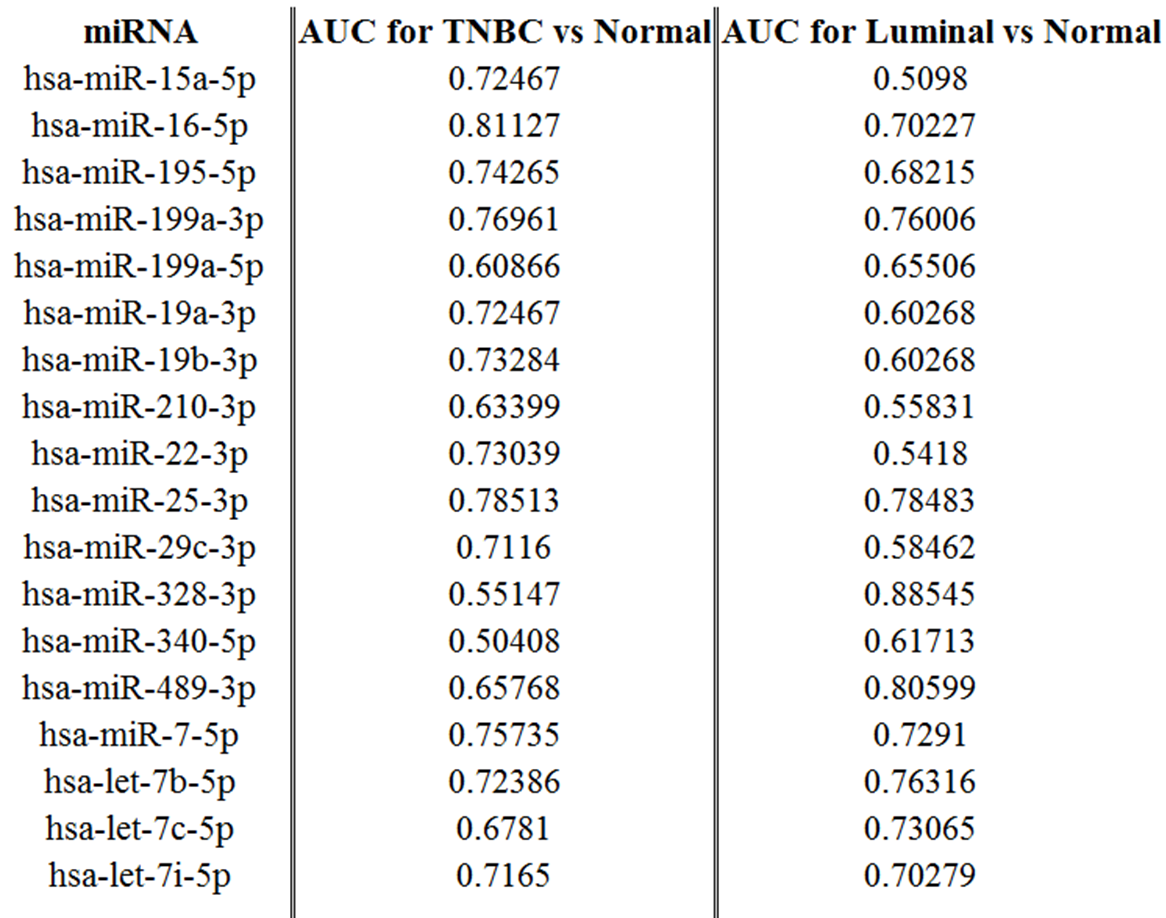
**

**Additional file 1: Table S2** Univariate AUC statistics for the differential miRNAs based on ROC Analysis
